# Complete genome sequence of *Bradyrhizobium japonicum* ACCC 15027

**DOI:** 10.1128/mra.00933-24

**Published:** 2025-12-17

**Authors:** Yuhan Zhang, Guohua Zou, Chao Li, Yongliang Yan, Min Lin, Yuhua Zhan

**Affiliations:** 1 Biotechnology Research Institute/National Key Laboratory of Agricultural Microbiology, Chinese Academy of Agricultural Sciences 12661, Beijing, China; 2 Biotechnology Research Institute/Key Laboratory of Agricultural Microbiome (MARA), Chinese Academy of Agricultural Sciences 12661, Beijing, China; 3 College of Agriculture, Henan University 12411, Kaifeng, Henan, China; University of Strathclyde, Glasgow, United Kingdom

**Keywords:** nodulation, legume symbionts, nitrogen fixation, *Bradyrhizobium*

## Abstract

Here, we report the complete genome sequence of *Bradyrhizobium japonicum* ACCC 15027 from the Agricultural Culture Collection of China (ACCC). The whole genome of ACCC 15027 is 10.02 Mb long and consists of a circular chromosome and two plasmids.

## ANNOUNCEMENT

*Bradyrhizobium japonicum* colonizes the root nodules of leguminous plants, where it enhances *Glycine max* (soybean) growth via biological nitrogen fixation (1). The diazotrophic capacity of *B. japonicum* has established its prevalence as a sustainable biofertilizer in modern agricultural practices (2). There are currently more than 100 publicly available genomes for *B. japonicum*. However, these strains exhibit genotypic and phenotypic variations (3). *B. japonicum* ACCC 15027 was purchased from the Agricultural Culture Collection of China (ACCC, http://www.accc.org.cn), and the identity was certified by ACCC. Characterizing the genome of ACCC 15027 will accelerate the development of new rhizobial inoculants.

ACCC 15027 was obtained as a lyophilized powder from ACCC. One hundred milligrams of freeze-dried culture was inoculated into 5 mL of yeast extract mannitol medium and then incubated at 30°C under aerobic conditions for 72 hours to restore its activity. Genomic DNA was extracted via the HiPure Soil DNA Mini Kit (Magen Biotechnology Co., Ltd., China).

For Illumina, we used a VAHTS Universal Plus DNA Library Prep Kit (Nanjing Vazyme Biotech Co., Ltd., China) for Illumina v.2 to construct the library. Sequencing was performed via the Illumina NovaSeq PE 150 platform at Azenta Life Sciences (Suzhou, China), producing 10,760,005 raw read pairs (Table 1). These reads were filtered and trimmed using Fastp v.0.23.0. The projected genome coverage is 21.37-fold.

**TABLE 1:** Sequencing, assembly, and annotation data

Features	Data
Illumina raw data	
No. of total bases	3,228,001,500
No. of paired reads	10,760,005
Average read length (nt)	150
Q20 content (%)	97.81
GC content (%)	63.45
PacBio raw data	
No. of total bases	214,635,504
No. of total sequences	40,952
Average sequence length	5,241.15
N50 read length (nt)	7,098
GC content (%)	63.45
Genomic features	
Genome size (bp)	10,022,871
GC content (%)	63.50, 59.56, 61.96
No. of protein-coding genes	9,293
No. of replicons	3
Replicon sizes (bp)	9,460,647; 440,193; 122,031
Plasmid identification	
Accession numbers of the matches	LN901634.1, AP014686.1
Identity (%)	99.13 and 99.96
Coverage (%)	17.90 and 26.67

**Fig 1:**
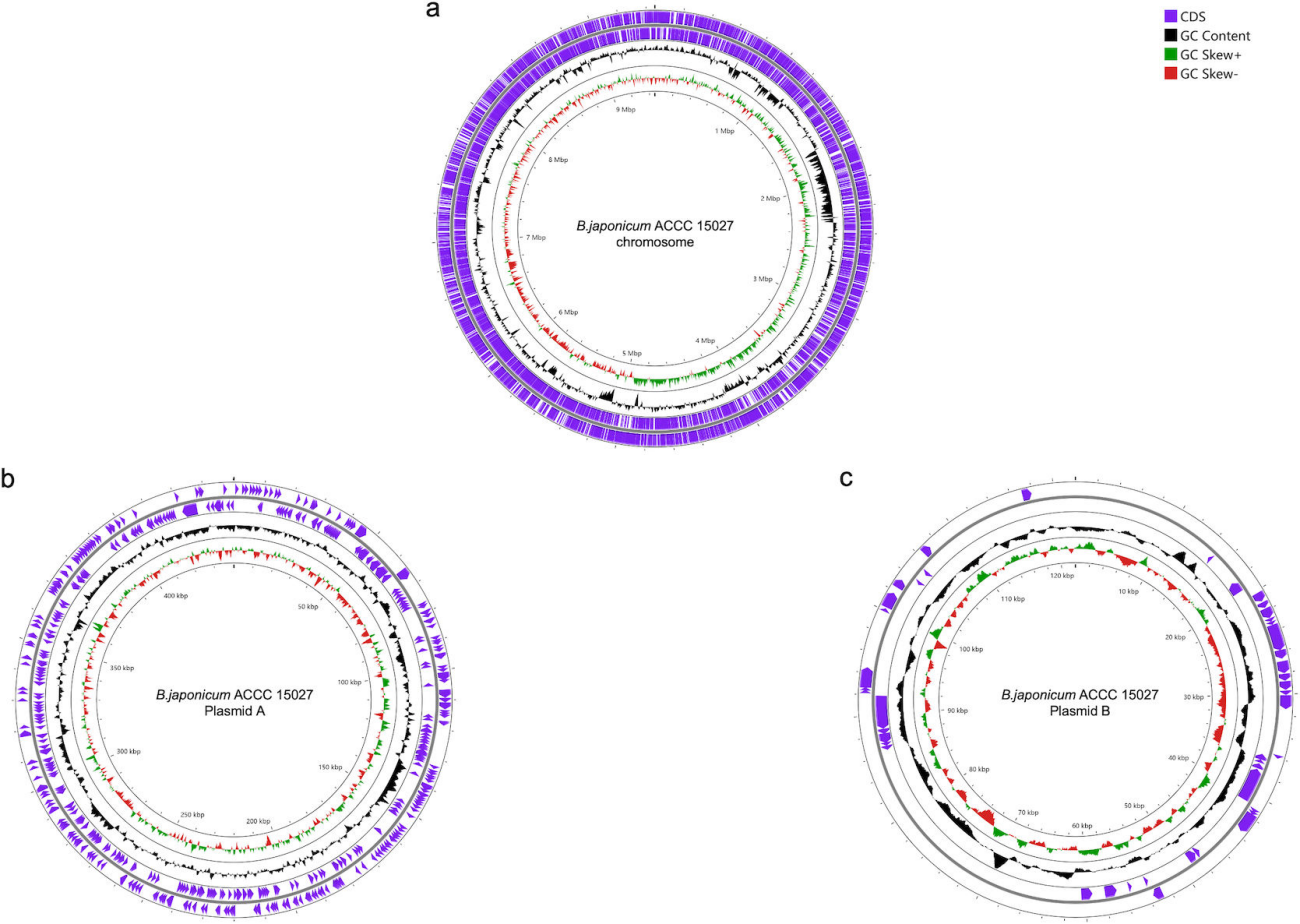
Circular map of the complete genome of *B. japonicum* ACCC 15027 generated via Proksee (https://proksee.ca/). The outer circle (purple) represents the coding sequence (CDS). The black circle indicates the GC content. The innermost circles (green and red) indicate the GC skew values, with green indicating a positive skew and red indicating a negative skew.

For the PacBio sequencing library, the same gDNA samples that were sequenced by Illumina were sheared into 10–15 kb fragments using a g-TUBE device (Covaris, USA). A library was subsequently constructed via the SMRTbell Express Template Preparation Kit 2.0 (Pacific Biosciences, USA). Sequencing was performed via “HiFi sequencing” on the platform PacBio Sequel II (Pacific Biosciences, USA) at Azenta Life Sciences (4). A total of 40,952 polymerase reads with an average read length of 5,241 bp were generated, and the N50 was 7,098 (Table 1). PacBio HiFi reads were assembled via Hifiasm v.0.19.5, resulting in three circular contigs (5). The genome was then corrected with Pilon v.1.22 software using the previous Illumina reads obtained in this study (6). Subsequently, the chromosome was reoriented using Circlator v.1.4.1 to start at the *dnaA* gene (7). The final assembly consists of one circular chromosome and two circular plasmids (plasmid A and plasmid B), with a total size of 10.02 Mb (Fig. 1). The chromosome size is 9.46 Mb, with a GC content of 63.68%.

The plasmid origin of the assembled contigs was determined through BLASTN v.2.17.0+ alignment against the NCBI NT database (8). Plasmid A and plasmid B were identified based on significant matches (99.13% identity and 99.96% identity, respectively) to known plasmids LN901634.1 and AP014686.1. Using NCBI PGAP v.6.10 (9), the whole genome was found to contain 9,226 protein-coding genes and 67 RNA genes. The average coverage and length were 84.20% and 878 bp, respectively. Unless otherwise noted, default parameters were used for all software.

Further analysis of the ACCC 15027 genome will contribute to the knowledge of economically important symbiotic bacteria, as well as to evolutionary and taxonomic research on the genus *Bradyrhizobium*.

## Data Availability

The complete genome sequence of *B. japonicum* ACCC 15027 was deposited in GenBank under accession numbers CP169752 (chromosome), CP169753 (plasmid A), and CP169754 (plasmid B); BioProject accession number PRJNA1159492; and BioSample accession number SAMN43576846. The raw sequence data are available under Sequence Read Archive accession numbers SRR31582189 (Illumina) and SRR30629247 (PacBio), respectively.
